# Validation of the Needs Assessment of Family Caregivers-Cancer scale in an Asian population

**DOI:** 10.1186/s40359-020-00445-x

**Published:** 2020-08-12

**Authors:** Winson Fu Zun Yang, Jianlin Liu, Yiong Huak Chan, Konstadina Griva, Sangita Kuparasundram, Rathi Mahendran

**Affiliations:** 1 National University Hospital, 5 Lower Kent Ridge Rd, Singapore, 119074 Singapore; 2 Department of Psychological Science, Texas Tech University, Box 42051, Lubbock, TX 79409-2051 USA; 3 Department of Psychological Medicine, Yong Loo Lin School of Medicine, Faculty of Medicine, National University of Singapore, Singapore, Singapore; 4 Institute of Mental Health, 10 Buangkok View, Buangkok Green Medical Park, Singapore, 539747 Singapore; 5 Dean’s Office, Yong Loo Lin School of Medicine, National University of Singapore, NUHS Tower Block, Level 11, 1E Kent Ridge Road, Singapore, 119228 Singapore; 6 Lee Kong Chian School of Medicine, Imperial College & Nanyang Technological University, 59 Nanyang Drive, Experimental Medicine Building, Singapore, 636921 Singapore; 7 SingHealth Residency, Ministry of Health Holdings, 1 Maritime Square, Singapore, 009253 Singapore; 8 Academic Development Department, Duke-NUS Medical School, 8 College Road, Singapore, 169857 Singapore

**Keywords:** Family caregivers, Caregiver needs, Cancer, Validation, NAFC-C

## Abstract

**Background:**

The Needs Assessment of Family Caregivers- Cancer (NAFC-C) scale is shown to have adequate psychometric properties in assessing family caregiver needs during the cancer journey and its psychometric properties have been studied only in Western populations. This study sought to validate the NAFC-C in an Asian population for wider applicability.

**Methods:**

Participants (*n* = 363) completed questions on sociodemographics, the Depression Anxiety Stress Scale, the Zarit Burden Interview, the Caregiver Quality of Life Index-Cancer scale, and the NAFC-C.

**Results:**

Results revealed good internal consistency, test-retest reliability, and concurrent validity of the NAFC-C. Confirmatory factor analysis did not demonstrate a good fit of the NAFC-C in our sample. Exploratory factor analysis revealed a similar factor structure in this study’s population. Further reliability and validity analyses with the EFA factor structure demonstrated similar reliability and validity assessments.

**Conclusions:**

The NAFC-C is shown to be applicable in an Asian population. It would be a useful instrument for determining family caregivers’ needs and to inform future interventions to address those needs and improve or maintain quality of life in both patients and their caregivers.

## Background

Family caregivers (FCGs) have a vital role in patient care throughout the cancer journey, particularly as patient care is increasingly transferred to ambulatory and home settings [1, 2]. In taking time off their daily routine to provide care, FCGs inevitably face unmet needs and experience distress in dealing with their care recipients’ symptoms and managing their psychological well-being, sometimes to an extent greater than what care recipients themselves face [3–6]. As FCGs’ needs affect the patient-caregiver relationship and quality of life, it becomes important to examine these needs to inform future interventions to improve quality of life in both patients and their caregivers [7, 8].

There has been a significant increase in the number of studies on cancer caregivers unmet needs, but these have been predominantly in western populations [9]. Several instruments have been developed to assess caregiver needs, such as the Cancer Caregiving Tasks, Consequences and Needs Questionnaire (CaTCoN) [10, 11], Cancer Survivors’ Partners Unmet Needs (CaSPUN) [12], Cancer Support Person’s Unmet Needs (SPUNS) [13], Comprehensive Needs Assessment Tool for Cancer-Caregivers (CNAT-C) [14], and the Needs Assessment of Family Caregivers- Cancer (NAFC-C) [15]. Both the SPUNS and the CNAT-C have been validated and translated for use in Chinese populations [16, 17]. However, only the NAFC-C, has been developed on a conceptual framework based on the Need Fulfillment Theory which enhances its clinical utility [18, 19].

The Need Fulfillment Theory suggests that people have physiological and socio-psychological needs which can be fulfilled and these needs determine and motivate how individuals act, think, or respond [20, 21]. Different levels of need fulfillment impact the relationship between satisfaction and importance, and a need is fulfilled when both importance and satisfaction are met [22, 23]. Need fulfillment is greatest at this point when the need is important and when the person is satisfied that the need is met. Conversely, need fulfillment is at its lowest when the need is largely unmet and associated with dissatisfaction. In cancer caregiving, FCGs experience needs in different dimensions such as psychological, social, medical, and financial needs [24]. Accordingly, the NAFC-C was designed to assess these important dimensions.

The NAFC-C measures four overarching factors: 1) psychosocial unmet needs, 2) medical unmet needs, 3) financial unmet needs, and 4) daily activity unmet needs. Each of these unmet needs include several sub-factors which provide a comprehensive evaluation of needs: 1) emotional distress, finding meaning, and relationship loading on the psychosocial unmet needs, 2) medical care, and symptom management on medical unmet needs, and 3) caregiver personal care, and balancing roles for survival care on the daily activity unmet needs. Additionally, these factors are rated on two important dimensions: the importance of the need, and the satisfaction with the fulfillment of the need during the past 4 weeks.

The NAFC-C had acceptable internal consistency (0.56 < αs < 0.86), and good divergent validity; its factors were able to predict quality of life through three different phases of cancer survivorship and across several dimensions of needs [15]. However, its psychometric properties have yet to be rigorously tested. The NAFC-C was validated in an educated and affluent Caucasian sample, hence the generalizability of the instrument to the community, and in non-Caucasian populations is unknown [15].

Cultural differences between Asian and Western societies affect caregiving patterns. Research suggests that Asians spend more time in caregiving compared to their Western counterparts and that their caregiving is ingrained with Asian ideologies such as filial piety and family obligations [25, 26]. Caregiving motivations also differ and have been identified as directed by personal value and fulfillment, care provision because of societal expectations, and practical need [27].

Validating the NAFC-C which incorporates both the need and degree of satisfaction of the need being met, in an Asian setting, would improve the assessment of caregiver needs and establish the strength of the instrument in this population. The study’s aim was to validate and establish the psychometric properties of the NAFC-C in Singapore, an island state in South East Asia populated by a predominantly English-speaking population consisting of multiethnic groups (Chinese, Malays, Indians) found in Asia. The secondary aim was to explore the factor structure of the NAFC-C.

## Methods

### Participants and procedure

FCGs (*N* = 517) of cancer patients (aged 21 and over) followed up in ambulatory clinics at the National University Cancer Institute Singapore, were invited to participate in this study; the recruitment period was from May 2017 to December 2017. Inclusion criteria were as follows: 1) Singapore citizens or permanent residents between 21 and 84 years of age, and 2) able to read and understand English.

Research assistants approached caregivers, accompanying their care recipients to the cancer clinic for appointments, and invited them to participate. Informed consent was taken in a separate private waiting room. Convenience sampling was used as caregivers and recipients were most accessible at clinics. While this is a non-probability sampling method and may not account for the type and stage of cancer, the top few cancers in Singapore, lung, breast, gastro-intestinal/colorectal/stomach, with the exception of prostate cancer, were adequately sampled (Singapore Cancer Registry).

Participants were asked to complete a questionnaire on socio-demography and their care recipient’s cancer diagnosis and treatment, and scales to assess their own mood state, quality of life, caregiving burden and needs (described in detail under Measures). Permission was received from the developer (Dr. YM Kim) to use and validate the NAFC-C in the local population. Participants were given a second NAFC-C questionnaire to complete 2 weeks later (Time 2) for validation purposes. Stamped and addressed envelopes were provided for the questionnaires to be mailed back; 405 questionnaires were returned. Of these, 42 were excluded from the analysis for the following reasons: they were not English speakers (*N* = 6), did not complete the NAFC-C (*N* = 25), were not family members (*N* = 9), withdrawal from the study (*N* = 1), and care recipient’s diagnosis being revised to ‘no cancer’ (N = 1). The final analysis was conducted on 363 participants who had completed the full set of the questionnaire at Time 1. Additionally, out of the 363 participants, only 233 participants completed the NAFC-C again at Time 2, and this subset of data was used to examine test-retest reliability. The study had Ethics Board approval (Reference No. 2017/000/29, Received: 25 April 2017), and written informed consent was obtained.

### Measures

#### Sociodemographic and medical characteristics

Participants completed a self-report questionnaire which collected two types of variables: 1) demographic variables comprising of age, sex, ethnicity, marital status, education, employment, income per capita, and identity of care recipient; and 2) medical variables of the care recipient comprising of type of cancer, cancer stage, and type and length of treatment and whether it was completed.

#### NAFC-C

The NAFC-C is a 27-item scale that measures different caregiver needs in the context of cancer care, on two dimensions: the importance of the need, and the satisfaction with the fulfillment of the need during the past 4 weeks [15]. Both dimensions are measured on a five-point Likert-type scale ranging from 0 (Not at all) to 4 (Extremely). Satisfaction rating was reverse coded for each item. For each item, needs score was computed by multiplying satisfaction with importance rating, yielding a range of 0 to 16, with a higher score indicating a higher index of unfulfillment. As mentioned, the scale consists of four factors 1) psychosocial unmet needs, 2) medical unmet needs, 3) financial unmet needs, and 4) daily activity unmet needs. Each unmet need also contains several sub-factors, with emotional distress, finding meaning, and relationship loading on the psychosocial unmet needs, medical care and symptom management loading on the medical unmet needs, and caregiver personal care and balancing roles for survival care loading on the daily activity unmet needs.

#### Depression anxiety stress scale (DASS)

The DASS-21 is a 21-item scale that measures depressive symptoms over the past week [28]. Items were measured on a four-point Likert-type scale ranging from 0 (Never) to 3 (Almost always). Total and subscale scores (depression, anxiety, and stress) were computed by summing up the items in their respective scale or subscale. Higher scores indicate higher depressive, anxiety, and stress symptoms. Good reliability and validity of this scale have been demonstrated in an Asian population [29]. The DASS reliability was demonstrated by good internal consistency for the overall scale (α = 0.95), depression (α = 0.90), anxiety (α = 0.85), and stress (α = 0.88) subscales, in this study sample.

#### Caregiver quality of life index-Cancer (CQOL-C)

The CQOL-C is a 35-item scale that measures the overall quality of life of cancer caregivers over the past week [30]. Items were measured on a five-point Likert-type scale ranging from 1 (Not at all) to 5 (Very much). Items 4, 10, 12, 16, 22, 27, 28, and 34 were reverse coded. Total score was computed by summing up all the items, with higher score indicating lower quality of life. The CQOL-C has shown good internal consistency, face, content, concurrent, convergent and also discriminant validity [30]. The CQOL-C has also been shown to be reliable and valid in a Singapore population and good internal consistency (α = 0.88) was further demonstrated in this sample [31].

#### Zarit burden interview (ZBI)

The ZBI is a 22-item scale that originally measured the burden experienced by caregivers of persons with dementia [32]. Items were measured on a five-point Likert-type scale ranging from 0 (Never) to 4 (Nearly always). Total score was computed by summing up all the items, with higher score indicating higher levels of burden experienced. While the ZBI was originally developed to assess burden experienced by caregivers of persons with dementia, it has now been widely used in many clinical contexts, such as Parkinson’s disease and chronic diseases, [33–36] and also shown to be reliable and valid for use in cancer caregivers [37, 38] and in an Asian population [39]. In this study, the ZBI reliability demonstrated good internal consistency (α = 0.93).

### Statistical analysis

#### Reliability

Internal consistency was assessed by analysing Cronbach’s alphas for the total NAFC-C scale and factors at both T1 and T2. A Cronbach’s alpha coefficient of more than 0.60 was considered acceptable [40, 41]. Test-retest reliability was assessed by calculating the correlation across time, and item-item correlations between the NAFC-C scores at both timepoints. The strength of correlations was based on Cohen’s criteria (weak: *r* = ±0.10 – ±0.29, moderate: ±0.30 – ±0.49, strong: *r* = ±0.50 – ±1.00) [42].

#### Validity

Concurrent validity was assessed correlating depressive symptoms (DASS), burden (ZBI), and quality of life (CQOL-C) scores on the NAFC-C. Covariates that were included in the analyses were age, sex, ethnicity, education, income, employment, and relationship with patient. Benjamini-Yekutieli correction (k = 30 comparisons, α = 0.01) was used to reduce the familywise Type I error rates [43]. The strength of correlations was based on Cohen’s criteria (weak: *r* = ±0.10 – ±0.29, moderate: ±0.30 – ±0.49, strong: *r* = ±0.50 – ±1.00) [42].

#### Model structure confirmation

Confirmatory factor analysis (CFA) was conducted to replicate the factor structure of the NAFC-C, consisting of four primary factors. Goodness-of-fit of the model was assessed based on the following fit indices with the recommended criteria: non-significant chi-square likelihood ratio test (χ^2^), comparative fit index (CFI ≥ 0.90), root-mean-square error of approximation (RMSEA ≤0.06), and standard root-mean-squared residual (SRMR ≤0.08) [44].

#### Model structure exploration

In an event where the CFA model was not a good fit, an EFA was conducted to explore the underlying factor structure in this study sample. Kaiser-Myer-Olkin (KMO) test and Bartlett’s test of sphericity were used to examine the data for sampling adequacy prior to conducting EFA. Sampling adequacy was based on a minimum KMO value of 0.50 and significant Bartlett’s test of sphericity [45]. Promax rotation was used to allow correlation between factors [46]. A scree plot of the eigenvalues, based on Catell’s criterion, was used to extract the optimum number of factors of the NAFC-C. Using that number (k), and a suitable rotation, a k-factor solution, was conducted [47]. Items that did not achieve a factor loading of at least 0.40 or cross-loadings of more than 0.30 on another factor were removed to ease interpretability of the results.

All analyses were conducted in in R 3.5.3 loading on R Studio 1.3.842. CFA and EFA were conducted using the *lavaan* package (v. 0.5–23.1097) [48].

## Results

### Demographics and clinical characteristics

Table 1 presents the socio-demographics and clinical characteristics of the participants. About three-fifths of the participants were female (*n* = 227, 62.50%), and almost all participants had at least secondary education (*n* = 347, 95.60%). The ethnicity closely paralleled the Singapore population. The most prevalent relationship with care recipients’ are parents (*n* = 169, 46.60%), followed by spouses (*n* = 116, 32.00%).

**Table 1:** Participants Demographics

Sociodemographic and medical variables	N (%^a^)
Sex	
Male	136 (37.50)
Female	227 (62.50)
Race	
Chinese	263 (72.50)
Malay	59 (16.30)
Indian	27 (7.44)
Others	12 (3.31)
Age Group (years)	
21–30	61 (16.80)
31–40	68 (18.70)
41–50	86 (23.70)
51–60	79 (21.80)
61–70	52 (14.30)
71–80	12 (3.31)
Education	
No formal education	2 (0.55)
Primary (Some/Completed)	12 (3.31)
Secondary (Some/Completed) / N, O Levels / ITE	104 (28.70)
A Levels / Poly Diploma	95 (26.20)
Bachelor’s Degree	116 (32.00)
Masters / PhD	32 (8.82)
Education (≥ High school)	
Yes	347 (95.60)
No	14 (3.86)
Marital Status	
Single	119 (32.80)
Married	221 (60.90)
Divorced/Separated	7 (1.93)
Widowed	2 (0.55)
Employed	
Yes	238 (65.60)
No	118 (32.50)
Income (per capita)	
$2000 & below	77 (21.20)
$2001 - $8000	166 (45.70)
$8001 & above	58 (16.00)
Relationship with care recipient	
Spouse	116 (32.00)
Parent	169 (46.60)
Grandparent	8 (2.20)
Son/daughter	19 (5.23)
Sibling	30 (8.26)
Others	1 (3.03)
Type of Cancer	
Breast	73 (20.10)
Lung	68 (18.70)
Gastro-intestinal/Colorectal/Stomach	59 (16.30)
Hemotological/Leukemia/Lymphoma/Myeloma	54 (14.90)
Gynecological	16 (4.41)
Pancreas	11 (3.03)
Multisite	12 (3.31)
NPC/Throat/Oral	13 (3.58)
Renal	8 (2.20)
Brain tumor	6 (1.65)
Cancer Stage if known	
Early (stages 0–2)	63 (17.40)
Late (stages 3–4)	247 (68.10)
Is treatment completed?	
No	265 (73.00)
Yes	83 (22.90)
Type of Treatment completed	
Chemotherapy	142 (39.10)
Radiotherapy	96 (26.40)
Surgery	147 (40.50)

^a^ Percentages might not sum up to 100% due to missing data, or rounding difference

### Reliability

The NAFC-C demonstrated good internal consistency at both T1 (α = 0.90) and T2 (α = 0.89) (Table 2). Cronbach’s alpha for each of the factors was also demonstrated to be good at T1 (α = 0.71–0.90) and T2 (α = 0.70–0.89). Excellent test-retest reliability was revealed for the total NAFC-C score (*r* = 0.80). Furthermore, all factors also demonstrated good test-retest reliability (*r* = 0.77–0.82) for all factors. Item-item correlations demonstrated acceptable to excellent correlation (*r* = 0.61–0.88) except for items 21 and 22, which demonstrated questionable test-retest reliability (*r* = 0.59 and *r* = 0.50 respectively; Table 3).

**Table 2:** Reliability of the Needs Assessment of Family Caregivers-Cancer (NAFC-C)

NAFC-C unmet needs	Original factor structure					EFA factor structure				
	Mean (SD)		Cronbach’s alpha		Test-retest	Mean (SD)		Cronbach’s alpha		Test-retest
	T1	T2	T1	T2		T1	T2	T1	T2	
Total	2.26 (1.69)	2.25 (1.66)	0.90	0.89	0.80	2.19 (1.63)	2.18 (1.60)	0.86	0.85	0.80
Psychosocial	2.09 (1.85)	2.06 (1.95)	0.79	0.81	0.77	1.83 (2.00)	1.91 (2.16)	0.76	0.81	0.75
Medical	3.07 (2.44)	3.07 (2.37)	0.78	0.75	0.80	3.36 (2.68)	3.60 (2.75)	0.78	0.76	0.84
Financial	1.82 (2.67)	1.92 (2.68)	0.71	0.70	0.77	1.76 (2.54)	1.75 (2.51)	0.78	0.77	0.76
Daily activity	1.89 (1.90)	1.86 (1.89)	0.80	0.80	0.82	1.90 (2.06)	1.72 (1.85)	0.80	0.72	0.75

**Table 3:** Test-retest reliability of the Needs Assessment of Family Caregivers-Cancer (NAFC-C) at timepoint 1 (T1) and timepoint 2 (T2)

Items	Description	r
Item 1	Helping his/ her emotional distress (e.g. anger, anxiety, depression, fear, resentment, etc.)	0.75
Item 2	Getting the best possible care for him/her	0.77
Item 3	Taking care of bills	0.76
Item 4	Meeting your personal needs	0.72
Item 5	Dealing with your emotional distress (e.g. anger, anxiety, depression, fear, resentment, etc.)	0.68
Item 6	Communicating with his/her medical staff	0.77
Item 7	Having enough insurance coverage for him/her	0.74
Item 8	Getting help from others in order to take time for yourself	0.78
Item 9	Talking to him/her about his/her concerns	0.74
Item 10	Getting involved in medical decisions affecting him/her	0.79
Item 11	Paying for his/her medical expenses	0.75
Item 12	Taking time off work	0.65
Item 13	Finding meaning out of your experience with his/her cancer	0.61
Item 14	Getting information about the cancer he/she was diagnosed with (e.g. prognosis, treatment, side effects, nutrition)	0.88
Item 15	Getting together with family and friends	0.65
Item 16	Helping him/her find meaning out of cancer	0.63
Item 17	Understanding/ Navigating medical and/or insurance coverage	0.66
Item 18	Being satisfied with your relationship with other family members and friends	0.67
Item 19	Managing his/her cancer-related pain	0.78
Item 20	Managing his/her other cancer-related symptoms (e.g. fatigue, nausea)	0.75
Item 21	Balancing work/school with caring for him/her	0.59
Item 22	Being satisfied with your relationship with him/her	0.50
Item 23	Reorganizing roles among family members	0.70
Item 24	Having an intimate relationship with him/her	0.63
Item 25	Dealing with lifestyle changes	0.70
Item 26	Assisting with his/her daily needs (e.g. preparing meals, transportation, etc.)	0.70
Item 27	Helping him/her adjust to life after cancer	0.76

### Validity

The NAFC-C demonstrated good concurrent validity via weak and positive correlations with the total (*r* = 0.27, *p* < 0.001), depression (*r* = 0.29, *p* < 0.001), anxiety (*r* = 0.19, *p* < 0.012), and stress (*r* = 0.26, *p* < 0.001) dimensions of the DASS, strong and positive correlations with the CQOL-C (*r* = 0.50, *p* < 0.001), and moderate and positive correlations with the ZBI (*r* = 0.48, *p* < 0.001; Table 4).

**Table 4:** Correlations between psychosocial measures and NAFC-C

Measures	Mean (SD)	Cronbach’s alpha	Original factor structure (r)					EFA factor structure (r)				
			Total	Psychosocial Unmet Needs	Medical Unmet Needs	Financial Unmet Needs	Daily Activity Unmet Needs	Total	Psychosocial Unmet Needs	Medical Unmet Needs	Financial Unmet Needs	Daily Activity Unmet Needs
DASS: Total score	11.10 (10.50)	0.95	0.27^***^	0.4^***^	0.01	0.18^**^	0.28^***^	0.24^***^	0.28^***^	0.09	0.24^***^	0.34^***^
DASS: Depression	3.43 (3.99)	0.90	0.29^***^	0.41^***^	0.05	0.17^**^	0.29^***^	0.25^***^	0.30^***^	0.06	0.23^***^	0.33^***^
DASS: Anxiety	3.03 (3.53)	0.85	0.19^**^	0.31^***^	0.06	0.20^**^	0.19^**^	0.18^**^	0.22^***^	0.15	0.25^***^	0.25^***^
DASS: Stress	4.76 (3.95)	0.88	0.26^***^	0.38^***^	0.03	0.12	0.29^***^	0.23^***^	0.26^***^	0.07	0.17^**^	0.34^***^
CQOL-C	53.50 (19.40)	0.88	0.50^***^	0.42^***^	0.39^***^	0.34^***^	0.42^***^	0.51^***^	0.36^***^	0.31^***^	0.36^***^	0.42^***^
ZBI	24.00 (14.40)	0.93	0.48^***^	0.47^***^	0.27^***^	0.28^***^	0.47^***^	0.47^***^	0.36^***^	0.19^**^	0.31^***^	0.47^***^

Table 4 displays the partial correlations between psychosocial measures and the NAFC-C in the original and new factor structure. Covariates that were included in the analyses were age, sex, ethnicity, education, income, employment, and relationship with patient. *NAFC-C* Needs Assessment of Family Caregivers-Cancer, *DASS* Depression Anxiety Stress Scale, *CQOL-C* Caregiver Quality of Life-Cancer, *ZBI* Zarit Burden Interview

^***^p < 0.001, ^**^p < 0.012

Further analyses on each NAFC-C factor revealed that the psychosocial unmet needs was moderately and positively correlated with the total (*r* = 0.40, *p* < 0.001), depression (*r* = 0.41, *p* < 0.001), anxiety (*r* = 0.31, *p* < 0.001), and stress (*r* = 0.38, *p* < 0.001) dimensions of the DASS, CQOL-C (*r* = 0.42, *p* < 0.001), and the ZBI (*r* = 0.47, *p* < 0.001).

Medical unmet needs was moderately and positively correlated with the CQOL-C (*r* = 0.39, *p* < 0.001), and weakly and positively with the ZBI (*r* = 0.27, *p* < 0.001).

Financial unmet needs was weakly and positively correlated with the total (*r* = 0.18, *p* < 0.001), depression (*r* = 0.17, *p* < 0.001), and anxiety (*r* = 0.20, *p* < 0.001) dimensions of the DASS, and the ZBI (*r* = 0.28, *p* < 0.001), and moderately and positively with the CQOL-C (*r* = 0.42, *p* < 0.001).

Daily activity unmet needs was weakly and positively correlated with the total (*r* = 0.28, *p* < 0.001), depression (*r* = 0.24, *p* < 0.001), anxiety (*r* = 0.17, *p* < 0.012), and stress (*r* = 0.24, *p* < 0.001) dimensions of the DASS, and moderately and positively with the CQOL-C (*r* = 0.36, *p* < 0.001), and ZBI (*r* = 0.34, *p* < 0.001).

### Model structure confirmation

The goodness-of-fit for the original model did not show a good fit (χ^2^ (318) = 1250.17, p < 0.001) based on the following statistics: CFI = 0.71, RMSEA = 0.09, and SRMR = 0.11 based on the recommended criteria (CFI ≥ 0.90; RMSEA ≤0.06; and SRMR ≤0.08) [44]. On further inspection, we reviewed the residual correlations. About 74 (21.08%) of all possible 351 residual correlations had r > 0.01, which may have caused the RMSEA to be poor, and thus a poor fit of the model.

### Model structure exploration

**Fig. 1:**
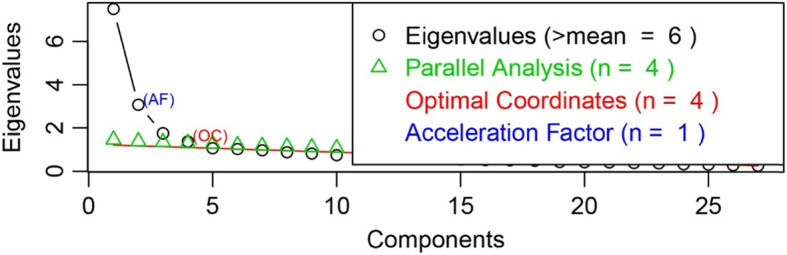
shows the scree plot of the exploratory factor analysis. Based on parallel analysis and Cattell’s criteria, 4 factors should be extracted from the data

As CFA did not show a good fit, an EFA was conducted to explore the factor structure of the NAFC-C. KMO measure of sampling adequacy (.89) and Bartlett’s test of sphericity (*χ*^*2*^(351) = 3698.58, p < 0.001) revealed that the sample size was adequate for EFA. A scree plot of the eigenvalues, based on Cattell’s criterion, revealed that the optimum number of factors of the NAFC-C was 4 (Fig. 1) [46, 47]. Hence, forcing a four-factor solution, and using a promax rotation, the EFA yielded four factors explaining a total of 42.66% of the variance for the entire set of variables. Items 1, 12,13, and 21 were removed because they did not have factor loadings greater than 0.40. Item 20 and 25 was removed because it had a cross-loading of more than 0.30 on another factor, and it loads similarly on two factors. Hence a second factor analysis was conducted with the removed items. The final four-factor solution had 21 items, which explained a total of 43.98% of the variance of the entire set of variables. The first, second, third, and fourth factors explained 12.06, 12.65, 9.28, and 10.99% of the variances respectively. Inspection of communalities showed that several items (items 3, 4, 5, 9, 10, 15, 19, 26) had low communalities (< 0.40; Table 5). Factor loadings of the NAFC-C is shown in Table 5.

**Table 5:** Factor loadings and communalities of the EFA factor structure of the NAFC-C

Items	Description	Original Factor loadings	EFA Factor loadings	communalities
*Factor 1: Daily activity*				
Item 4	Meeting your personal needs	0.59	0.63	0.37
Item 5^a^	Dealing with your emotional distress (e.g. anger, anxiety, depression, fear, resentment, etc.)	0.58	0.51	0.37
Item 8	Getting help from others in order to take time for yourself	0.62	0.77	0.52
Item 15	Getting together with family and friends	0.55	0.50	0.31
Item 18	Being satisfied with your relationship with other family members and friends	0.62	0.69	0.48
Item 23	Reorganizing roles among family members	0.69	0.51	0.44
*Factor2: Medical*				
Item 2	Getting the best possible care for him/her	0.59	0.62	0.41
Item 6	Communicating with his/her medical staff	0.61	0.78	0.54
Item 9^a^	Talking to him/her about his/her concerns	0.38	0.42	0.29
Item 10	Getting involved in medical decisions affecting him/her	0.59	0.62	0.39
Item 14	Getting information about the cancer he/she was diagnosed with (e.g. prognosis, treatment, side effects, nutrition)	0.67	0.77	0.59
*Factor 3: Financial*				
Item 3	Taking care of bills	0.67	0.57	0.30
Item 7	Having enough insurance coverage for him/her	0.62	0.69	0.50
Item 11	Paying for his/her medical expenses	0.73	0.68	0.50
Item 17^b^	Understanding/ Navigating medical and/or insurance coverage	0.35	0.74	0.61
*Factor 4: Psychosocial*				
Item 16	Helping him/her find meaning out of cancer	0.62	0.54	0.43
Item 19^b^	Managing his/her cancer-related pain	0.58	0.49	0.39
Item 22	Being satisfied with your relationship with him/her	0.66	0.55	0.44
Item 24	Having an intimate relationship with him/her	0.61	0.65	0.45
Item 26^c^	Assisting with his/her daily needs (e.g. preparing meals, transportation, etc.)	0.44	0.48	0.33
Item 27	Helping him/her adjust to life after cancer	0.52	0.71	0.49
*Items removed in the EFA-NAFC-C*				
Item 1^a^	Helping his/ her emotional distress (e.g. anger, anxiety, depression, fear, resentment, etc.)	0.40	–	–
Item 12^c^	Taking time off work	0.47	–	–
Item 13^a^	Finding meaning out of your experience with his/her cancer	0.42	–	–
Item 20^b^	Managing his/her other cancer-related symptoms (e.g. fatigue, nausea)	0.70	–	–
Item 21^c^	Balancing work/school with caring for him/her	0.66	–	–
Item 25^a^	Dealing with lifestyle changes	0.74	–	–

^a^. Items belong to psychosocial unmet needs in the original factor structure

^b^. Items belong to medical unmet needs in the original factor structure

^c^. Items belong to daily activity unmet needs in the original factor structure

*NAFC-C* Needs Assessment of Family Caregivers-Cancer

#### Reliability of the EFA factor structure

Further reliability and validity analyses were conducted to explore the psychometrics of the EFA factor structure. The NAFC-C (EFA) demonstrated good internal consistency at both T1 (α = 0.86) and T2 (α = 0.85) (Table 2). Cronbach’s alpha for each of the factors was also demonstrated to be good at T1 (α = 0.76–0.86) and T2 (α = 0.72–0.85). Excellent test-retest reliability was revealed for the total NAFC-C score (r = 0.80). Furthermore, all factors also demonstrated good test-retest reliability (r = 0.74–0.84).

#### Validity of the EFA factor structure

The NAFC-C demonstrated good concurrent validity via weak and positive correlations with the total (*r* = 0.24, *p* < 0.001), depression (*r* = 0.25, *p* < 0.001), anxiety (*r* = 0.18, *p* < 0.012), and stress (*r* = 0.23, *p* < 0.001) dimensions of the DASS, strong and positive correlations with the CQOL-C (*r* = 0.51, *p* < 0.001), and moderate and positive correlations with the ZBI (*r* = 0.47, *p* < 0.001; Table 4).

Further analyses on each NAFC-C factor revealed that the psychosocial unmet needs was weakly and positively correlated with the total (*r* = 0.28, *p* < 0.001), anxiety (*r* = 0.22, *p* < 0.012), and stress (*r* = 0.26, *p* < 0.001) dimensions of the DASS, and moderately and positively correlated with the depression dimension of the DASS (*r* = 0.30, *p* < 0.001), CQOL-C (*r* = 0.36, *p* < 0.001), and the ZBI (*r* = 0.36, *p* < 0.001).

Medical unmet needs was moderately and positively correlated with the CQOL-C (*r* = 0.31, *p* < 0.001), and weakly and positively with the ZBI (*r* = 0.19, *p* < 0.012).

Financial unmet needs was weakly and positively correlated with the total (*r* = 0.24, *p* < 0.001), depression (*r* = 0.23, *p* < 0.001), anxiety (*r* = 0.25, *p* < 0.001), and stress (*r* = 0.17, *p* < 0.012) dimensions of the DASS, and moderately and positively with the CQOL-C (*r* = 0.42, *p* < 0.001) and ZBI (*r* = 0.31, *p* < 0.001).

Daily activity unmet needs was moderately and positively correlated with the total (*r* = 0.34, *p* < 0.001), depression (*r* = 0.33, *p* < 0.001), and stress (*r* = 0.34, *p* < 0.001) dimensions of the DASS, the CQOL-C (*r* = 0.42, *p* < 0.001), and ZBI (*r* = 0.47, *p* < 0.001), and moderately and positively with the anxiety (*r* = 0.25, *p* < 0.001) dimension of DASS.

On further inspection, some items were loaded on another factor in the EFA factor structure (Table 5). Items 5 and 9 were in the psychosocial unmet needs in the original NAFC-C, but loaded onto daily activity and medical unmet needs respectively in the EFA factor structure. Furthermore, items 17 and 19 originally loaded onto medical unmet needs, but loaded onto financial and psychosocial unmet needs respectively in the EFA factor structure. Finally, items 26 originally loaded onto daily activity unmet needs, but loaded onto psychosocial unmet needs in the EFA factor structure.

Overall, the validities of the NAFC-C (EFA) mimics the validities of the original NAFC-C in terms of their strengths of association. However, there are some subtle differences, in which the NAFC-C (EFA) had weaker associations between psychological unmet needs and DASS, CQOL-C, and ZBI, and stronger association between medical unmet needs and CQOL-C and ZBI compared to the original NAFC-C. Other associations were of similar range and strengths.

## Discussion

This study demonstrated that the NAFC-C is reliable in an Asian population. It also showed that the NAFC-C has good criterion validity, although the original factor structure does not seem to be a good fit in this population.

### Psychometric properties of the original NAFC-C

The psychometric properties of the NAFC-C found in this Asian population were similar to the United States (US) population [15]. The internal consistency of the NAFC-C (α = 0.71–0.90) is consistent with the original across multiple cohorts (α = 0.66–0.86), although Cronbach’s alphas were higher in this sample [15]. While the internal consistency for financial unmet needs was the lowest in the local population, this was similar to the US sample, reflecting the consistency of the scale in different populations. This study found strong agreement between NAFC-C scores at T1 (α = 0.80) and T2 (α = 0.80), demonstrating good test-retest reliability.

Concurrent validity of the NAFC-C was supported by the weak to moderate positive correlations with the DASS, CQOL-C, and ZBI. Highest correlations were found between these measures and mental unmet needs (psychosocial and daily activities), while lowest correlations were found between physical unmet needs (medical and financial).

Low correlations found between DASS and physical unmet needs may be due to the healthcare infrastructure in Singapore. The availability of financial assistance is known to mitigate the psychosocial impact on caregivers and their patients [49]. In Singapore, financial support for healthcare in terms of subsidized fees for citizens and permanent residents seeking medical care helps to mitigate the financial strain on healthcare consumers [50]. Interestingly, we found near-zero correlations between medical unmet needs and DASS. Medical needs require caregivers to be involved in medical decisions regarding their care recipients, communicating with the healthcare providers, finding relevant medical information, and managing cancer-related symptoms and pain amongst other needs [15]. While the non-association may seem counterintuitive, a study conducted in South Korea [51] found that severe depression was associated with unmet medical needs. In our study sample, most of the participants in our sample did not have symptoms of depression, and were within normal functioning or mild. Therefore, this may have contributed to the low- or near-zero correlations between depression symptoms and medical unmet needs. Furthermore, cancer care in Singapore often involves patients and family members in medical consultations and support is extensive throughout the cancer journey [52, 53]. Furthermore, in Singapore and many Asian societies, major decision-making instances often involve family members as family members often feel responsible as part of the family unit (further discussed below) [52]. Together with the confidence in healthcare providers [54], these factors may have mitigated the association between FCG medical unmet needs and depression symptoms.

The results paralleled earlier work in which quality of life was associated with various unmet needs [15]. A previous study also found similar results, in which mental needs were associated with psychosocial distress (depression, anxiety, stress, and burden), and medical and financial needs were associated with quality of life but not psychosocial distress [55]. This is similar to our study where the association between physical unmet needs and psychosocial distress were not as strong as psychological unmet needs (psychosocial and daily activity) and psychosocial distress (DASS).

### Psychometric properties of the NAFC-C (EFA)

The EFA conducted suggested a similar structure from the original NAFC-C [15]. While each factor consists of items mostly from the original structure, there were some items that were from another factor in the original NAFC-C. This was not surprising and could be due to cultural and linguistic differences in Asia [56]. For example, item 9 (talking to him/her about his/her concerns) was perceived as medical-related concerns rather than psychological concerns. Patients in Asia are more likely to focus on medical symptoms in a healthcare setting than raise psychological concerns. Conceptualization of mental illness in Singapore, with a predominantly Chinese population, may have been influenced by concepts of Traditional Chinese Medicine. The Chinese believe that mental distress is a result of imbalance in the bodily functions [57]. They express psychosocial complaints as physical or medical complaints [58]. Hence this could explain why FCGs associate item 9 with medical needs rather than psychosocial needs.

In another example, item 17 (Understanding/ Navigating medical and/or insurance coverage) was observed to be associated with the financial domain than the medical domain, and it was highly correlated with item 7 (Having enough insurance coverage for him/her). Furthermore, insurance coverage is predominantly a financial concern for many patients and family members [59]. Hence item 17 was more suited to be in the financial rather than medical domain in our sample.

Given attitudinal differences towards medical or psychosocial symptoms in Asia, beliefs on pain and psychosocial symptoms could be understood the same way. In Asia, pain has many psychosocial connotations, and hence cancer-related pain (item 19) could be understood as psychosocial in Singapore [60]. Participants may have interpreted “Managing his/her cancer-related pain” as managing the psychological aspects of physical pain such as fear and anxiety. In the Asian cultural context, pain has a “welding” essence because the physical and mental aspects of pain are interrelated and not differentiated [61]. Specifically, it contains two Chinese characters, Tong-ku (Chinese phonetic), or 痛苦 (Chinese characters). While the first character denotes pain and the second character denotes bitterness, pain is not limited to only physical pain, but also mental and existential pain [55]. On the other hand, the “bitterness” usually denotes some form of hardship or suffering. Therefore, pain, in Asian cultures like Singapore, often depicts the coexistence of physical, mental, and existential elements of pain and suffering. Consequently, it is unclear whether participants interpreted cancer-related pain as physical, mental, or existential. Further research into family members’ perception of their care recipients’ pain should be conducted in order to create better instruments that capture these factors. For instance, phenomenological research could be conducted to understand caregivers’ concerns in greater detail, in the same way patients’ concerns were understood [62].

Another reason that could explain this difference is the low communalities of these items in the EFA, suggesting that these items may not be suitable in the NAFC-C in Singapore’s context [41]. Furthermore, on further inspection of the factors, correlations between daily activity and psychosocial unmet needs is strong (r = 0.61) compared to correlations between other factors (0.16–0.48). Hence, the shared variance between daily activity and psychosocial unmet needs might obscure some of the items, especially items 5 and 26, which load on opposite factors respectively. While item 5 belonged to psychosocial unmet needs in the original NAFC-C, it loaded on daily activity unmet needs in the EFA model. On the other hand, item 26 loaded on psychosocial unmet needs although it belonged to daily activity unmet needs. These items do not possess strong face validity in the EFA model as they clearly tap on the factors in the original structure, i.e., item 5 in psychological unmet needs, item 26 in daily activity unmet needs, rather than the ones in the our EFA structure, i.e., daily activity and psychological unmet needs respectively. Further inquiry into these items should be taken, for example, collecting qualitative data on these items, removing these items, or adding more items of similar nature.

It is also important to note that the total variance of unmet needs explained by the EFA model was only 43.98%. One reason for this may be that the items in the NAFC-C may not fit well in our sample. This was demonstrated by low communalities in some of the items. For some items, the factor loadings also differ between the NAFC-C (EFA) and original NAFC-C, which adds to the lack of item fit for the NAFC-C in our sample. Furthermore, the cultural differences of our sample participants may have led to different interpretations for Western counterpartsgiving rise to lower item fit in the model. Future studies could employ item response theory to exmine item fit in the model to provide additional psychometric evidence about the item characteristics on the NAFC-C [63]. Additionally, more items that identify with the Asian culture could be added into the NAFC-C to further investigate an Asian-variant of the scale. This may further provide sound psychometric support for the scale. A second reason may be that the spectrum of needs may not have been captured well with the current items. More items that capture the variability and dimension of needs may help to improve the factor structure and hence the total variance explained. Future study may need to add more items that are identifiable with the Asian culture.

### Common psychometric properties in both original and EFA-NAFC-C

A remarkable finding in the original and EFA-NAFC-C was the weak correlation between the NAFC-C and DASS. Cultural and linguistic differences between the two populations could have influenced how items were interpreted, and the choices made by participants [64, 65]. Oei and colleagues (2013) found a different factor structure of the DASS in an Asian population, but there were no comparisons with US populations on the items [29]. It is also possible that FCGs in Singapore could have experienced different needs. As correlations were weak in both the original and NAFC-C (EFA), caution must be exercised in interpreting these results.

Additionally, compared to quality of life and burden, depressive symptoms have lower correlations with the NAFC-C. This may be due to the cultural connotations of caregiving in Asian cultures like Singapore. In Asian societies, filial piety and family obligations are important, especially when a family member is ill. Although this may be the sole motivation for their caregiving, they may still face emotional distress, but chose not to disclose or express them [27]. They may endure emotional distress due to perceived responsibility, obligation, love, and duty of care to their family members. This was found in a recent study where caregivers’ depressive symptoms were low and stable compared to quality of life and burden across the first year post-diagnosis of cancer in their care recipients as they were mitigated by cultural expectations of family obligations and filial piety [66].

### Implications of the use of NAFC-C in an Asian society

The validation of an instrument like the NAFC-C, has several important implications in Singapore. Caregiving culture in Singapore is heavily based on filial piety and family obligations; caregivers may feel uneasy if their care recipient was in pain and they could not help to ease the suffering or pain [26]. By spending more time in caregiving, FCGs risk facing higher burden and lower quality of life [25, 66].

Second, Singapore’s healthcare system evolves continually to improve medical care; a recent forum on cancer supportive and survivorship care for example, focused on community and integrated cancer care, amongst other important factors discussed [67]. Additionally, the healthcare system ensures financial support for those seeking medical care and together with the family-centered approach in care delivery, physical needs such as medical and financial needs may not be an issue for caregivers unlike psychological needs such as psychosocial and daily activity needs [50, 52].

Finally, the perception of some items or constructs have deep cultural influence in Singapore. For example, in Asian culture, pain has multidimensional elements, specifically physical, mental, and existential [61]. These elements are interrelated and ingrained in Asian culture; hence needs such as pain may not fit perfectly in either medical or psychosocial unmet needs in Singapore. While the NAFC-C may still be relevant in Singapore, caution must be taken when interpreting these items. We advise researchers and clinicians to adopt the NAFC-C (EFA) in the Asian context, specifically in countries similar to Singapore, such as Hong Kong.

### Limitations

One methodological concern of this study is that with the small size in each cohort there was insufficient power to conduct cohort analysis or review psychometric properties of the NAFC-C at different phases of the cancer journey. This study was also unable to conduct a split-half EFA and CFA with the same sample due to a small sample size (*N* = 363). Hence the results in this study should be taken with caution. Future work should investigate this further in a larger sample. A second limitation is that no further analyses were conducted on the eight sub-factor structure which was found to have a poor fit in this sample. Due to the exploratory nature of the factor analysis conducted in this work, further rigorous testing on the structure and validity such as discriminant validity of the NAFC-C (EFA) should still be conducted in an independent sample. A third limitation is the short time interval between test and re-test. The heterogeneity of disease and treatment trajectories, may affect caregivers’ unmet needs and a longer test-retest interval for at least a month may capture the test-retest reliability better.

## Conclusion

The NAFC-C is based on sound theoretical foundations, and future work should investigate the needs of FCGs longitudinally for insights on the different needs of FCGs as they progress along the cancer continuum. As people in Asia speak different languages, validation of this questionnaire in Asian languages, would be crucial for clinicians, researchers, and policy makers. In particular, the translation and validation of a simplified Mandarin version of the NAFC-C would benefit researchers in parts of Asia such as China. where this text is used. It would allow the capture of FCGs concerns more accurately if done in their own mother tongue.

In conclusion, this study showed that the NAFC-C is reliable and valid in an Asian population and was associated with measures of depression, burden and quality of life. It is an appropriate psychometric tool to understand the needs of family members of cancer patients in an Asian population and would support the development of FCG interventions based on their needs and enable further research into association studies between risk factors and needs.

Although the original NAFC-C and NAFC-C (EFA) have similar factor structure, the NAFC-C (EFA) appears to closely identify with Asian culture. Therefore, researchers and clinicians in Asia could adopt the NAFC-C (EFA).

## Data Availability

The data is available from the corresponding author on reasonable request and subject to Ethics Board approval.

## Abbreviations

CaSPUN: Cancer Survivors Partners Unmet Needs
CaTCoN: Cancer Caregiving Tasks, Consequences and needs Questionnaire
CFA: Confirmatory Factor Analysis
CFI: Comparative Fit Index
CNAT-C: Comprehensive Needs Assessment Tool for Cancer-Caregivers
CQOL-C: Caregiver Quality of Life Index-Cancer
DASS: Depression Anxiety Stress Scale
EFA: Exploratory Factor Analysis
KMO: Kaiser-Myer-Olkin test
NAFC-C: Needs Assessment of Family Caregivers-Cancer
RMSEA: Root-mean-square error of approximation
SPUNS: Cancer Support Person’s Unmet Needs
ZBI: Zarit Burden Interview
